# Breaking Down Barriers: CorA Effectively Targets Staphylococcal Biofilms in Vitro and in Vivo

**DOI:** 10.1002/cmdc.202501057

**Published:** 2026-04-14

**Authors:** Stefania De Benedetti, Nikolay Krasimirov Kirilov, Haoxuan Zeng, Carolin Ade, Isabel Bodenstein, Maximilian Förster, Noran Abdel‐Wadood, Ahmad Aljohmani, Gabriela Krasteva‐Christ, Daniela Yildiz, Sabryna Junker, Syeda Javariya Khalid, Katharina Rox, Hedda Schrey, Miriam Große, Andrea Schiefer, Sören Leif Becker, Kenneth Pfarr, Achim Hoerauf, Markus Bischoff, Mathias Müsken, Tanja Schneider

**Affiliations:** ^1^ Institute for Pharmaceutical Microbiology University Hospital Bonn Bonn Germany; ^2^ German Centre for Infection Research DZIF Partner site Bonn‐Cologne Bonn Germany; ^3^ Institute for Medical Microbiology and Hygiene Saarland University Homburg Germany; ^4^ Department of Microbial Drugs Helmholtz Centre for Infection Research Braunschweig Germany; ^5^ Institute of Microbiology Technical University of Braunschweig Braunschweig Germany; ^6^ Institute of Anatomy and Cell Biology Saarland University Homburg Germany; ^7^ Preclinical Center for Molecular Signaling Molecular Pharmacology Saarland University Homburg Germany; ^8^ Helmholtz Centre for Infection Research Helmholtz Institute for Pharmaceutical Research Saarland Braunschweig Germany; ^9^ Department of Chemical Biology Helmholtz Centre for Infection Research Braunschweig Germany; ^10^ German Center for Infection Research DZIF Partner site Hannover‐Braunschweig Braunschweig Germany; ^11^ Institute of Medical Microbiology Immunology and Parasitology University Hospital Bonn Bonn Germany; ^12^ Central Facility for Microscopy Helmholtz Center for Infection Research Braunschweig Germany

**Keywords:** antibiotics, biofilms, corallopyronin a, drug discovery, *staphylococcus aureus*

## Abstract

Biofilm‐associated infections caused by *Staphylococcus aureus* (*S. aureus*) remain notoriously difficult to treat due to their pronounced tolerance to most antibiotics. Here, we evaluated the antibiofilm efficacy of the natural product antibiotic corallopyronin A (CorA) across a panel of strains, including clinically relevant strains differing in their biofilm‐forming capacities and antibiotic resistance profiles. CorA is an alpha‐pyrone antibiotic produced by *Corallococcus coralloides*. It targets the switch region of the bacterial DNA‐dependent RNA polymerase, thereby blocking transcription initiation at a site distinct from the rifampicin‐binding pocket, and displays potent activity against staphylococci, including MRSA and rifampicin‐resistant *S. aureus*. In vitro, CorA eradicated and inhibited biofilm formation, outperforming the biofilm‐active antibiotics dalbavancin and rifampicin both in optical density measurements and in microscopic analyses. Importantly, CorA had activity against rifampicin‐resistant strains in these assays. In a murine foreign body infection model with *S. aureus* SA113, CorA treatment resulted in *a* > 4‐*log*_10_ reduction in bacterial loads on implanted devices and surrounding tissues, comparable with high‐dose rifampicin, and significantly reduced local inflammation. These findings position CorA as a promising candidate for preventing and managing staphylococcal biofilm‐associated infections, warranting further investigation into its clinical potential.

## Graphical abstract

Corallopyronin A (CorA), a natural alpha‐pyrone antibiotic, penetrates *Staphylococcus aureus* biofilms and inhibits RNA polymerase. This work demonstrates CorA's superior efficacy compared to rifampicin and dalbavancin in reducing viable loads in biofilms both in vitro and in vivo. Graphic created in https://BioRender.com.

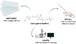

## 1 Introduction

*Staphylococcus aureus* (*S. aureus*), an opportunistic pathogen capable of causing diseases ranging from superficial skin infections to life‐threatening conditions, remains a leading cause of morbidity and mortality worldwide [1, 2]. Together with coagulase‐negative staphylococci (CoNS), it represents one of the most frequent causes of biofilm‐associated infections in clinical practice [3, 4]. Bacterial biofilms, structured communities embedded in a matrix of polysaccharides, extracellular DNA, and proteins, provide bacteria with enhanced protection against both host immune defenses and most antibiotics, thereby driving chronic, recurrent, and difficult‐to‐treat infections such as endocarditis, osteomyelitis, prosthetic joint infections, and diabetic foot ulcers [5, 6, 7, 8]. It is estimated that up to 80% of chronic bacterial infections involve biofilms [6]. These infections are notoriously persistent, often requiring prolonged antimicrobial therapy or surgical intervention, including device removal [9]. They impose a substantial clinical and economic burden due to high recurrence rates, increased morbidity, and longer hospital stays [4]. The management of staphylococcal biofilm infections is further complicated by the rising prevalence of multidrug‐resistant strains [10]. Standard therapies, usually involving rifampicin‐based combination regimens with glycopeptides (vancomycin, dalbavancin), remain the mainstay for staphylococcal biofilm infections, but glycopeptides exhibit limited activity once biofilms are established [11, 12]. Rifampicin, while considered a cornerstone in biofilm therapy, is compromised by hepatotoxicity [13, 14], its drug–drug interaction potential, and the rapid selection of resistance [15, 16]. Consequently, there is an urgent need for novel agents with improved activity against both planktonic and biofilm‐associated staphylococci.

Corallopyronin A (CorA) is a natural product produced by *Corallococcus coralloides* [17]. It acts by inhibiting bacterial RNA polymerase at the switch region, a site distinct from the rifampicin‐binding pocket. This unique mode of action confers activity against rifampicin‐resistant (Rif‐R) strains while sparing eukaryotic RNA polymerases [18]. CorA exhibits potent antibacterial activity against a broad spectrum of Gram‐positive pathogens, including methicillin‐resistant *S. aureus* (MRSA), as well as selected Gram‐negative species lacking efflux pumps, and bacterial endosymbionts such as *Wolbachia* [19]. The frequency of resistance development to CorA was lower than that observed for rifampicin, with CorA‐resistant mutants harboring mutations in *rpoB* or *rpoC* [18]. CorA has favorable ADME properties with a lower drug–drug interaction potential compared to rifampicin [20]. Additionally, CorA has excellent tissue penetration in bone [21] and penetrates well into host cells [22]. It is currently in preclinical development as a treatment for filarial nematode infections by targeting their endosymbiont *Wolbachia*.

Here, we investigated the activity of CorA against *S. aureus* biofilms, both in in vitro and in vivo settings. Together with its potent antibiofilm activity and low frequency of resistance development [18], these properties support CorA as a promising candidate for the treatment of biofilm‐associated infections, a clinical setting in which conventional antibiotics frequently fail [23].

## 2 Results and Discussion

### 2.1 CorA Inhibits Biofilm Formation and Eradicates Established Biofilms at MIC‐Level Concentrations

Initial crystal violet screenings of nine *S. aureus* reference strains, including ATCC 25923, HG001, and the osteomyelitis isolate ATCC 53657‐A, as well as 64 clinical *S. aureus* and coagulase‐negative staphylococcal (CoNS) isolates from prosthetic joint infections, confirmed a broad variability of biofilm formation phenotypes within the genus *Staphylococcus*. Among the tested strains, 28 were classified as strong biofilm formers, exhibiting absorbance values exceeding 3. Twenty‐one strains demonstrated moderate biofilm formation, with absorbance values between 2 and 3, whereas another 21 strains were identified as weak biofilm formers, displaying absorbance values below 2 (Supporting Information Table S1). From this strain collection, 15 representative strains were selected for detailed investigation (Supporting Information Table S1), based on their moderate‐to‐strong biofilm‐forming ability, resistance profiles [24], and diversity in MIC and MBC values [25], thereby covering a representative spectrum of biofilm phenotypes.

**FIGURE 1:**
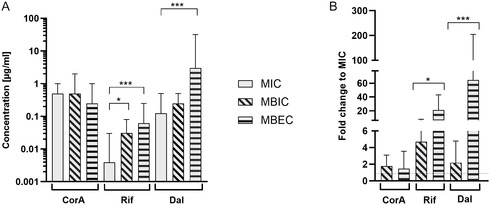
(A) Comparison between MIC (solid), MBIC (diagonal lines), and MBEC (horizontal lines) values for CorA, rifampicin (Rif), and dalbavancin (Dal). All experiments were performed in TSB containing 0.5% glucose. MIC represent the minimal inhibitory concentration of the compounds on planktonic cells. For the MBIC determinations, compounds were added to the wells at the beginning of the experiment and bacterial inocula were incubated for 24 h at 37°C under static conditions. For the MBEC, biofilms were allowed to form for 24 h prior antibiotic challenge. *n* = 15 strains, with a minimum of three biological replicates. The median and range is shown. *, *p* < 0.05; ***, *p* < 0.0001 (Kruskal–Wallis test followed by Dunn's post hoc test). (B) Fold changes of the MBIC and MBEC in relation to the MIC of a given strain. Dotted line represents 1‐fold. Data shown represent the mean and SD. *, *p* < 0.05; ***, *p* < 0.0001 (Mann–Whitney U test).

The crystal violet assay [26], chosen for the initial screening, stains total surface‐associated biomass but cannot discriminate between live and dead bacteria within the biofilm matrix and, thus, may overestimate residual biomass during eradication studies. For this reason, the quantitative determination of the minimal biofilm inhibitory concentration (MBIC) and minimal biofilm eradication concentration (MBEC) was performed using the Calgary biofilm device, a commercially available, standardized peg‐lid system that allows controlled biofilm growth and antibiotic exposure under identical conditions across multiple replicates [27]. In this system, the MBIC and MBEC are determined either by quantifying CFU/mL, using an agar spot assay, or by measuring the optical density (OD_600_) of the cell pools recovered from the pegs directly in a 96‐well plate in relation to a drug‐free control. Validation experiments confirmed that results derived from optical density readouts were consistent with the CFU‐based ones (max one 2‐fold dilution step difference, Supporting Information Figure S1), confirming the robustness and reproducibility. For the MBIC, compounds were added with the inoculum in the plate and the biofilm was allowed to grow under antibiotic challenge for 24 h. For the MBEC determination, biofilms were allowed to grow on the Calgary peg lids for 24 h and were subsequently exposed to antibiotics for 24 h. These experiments revealed that CorA and dalbavancin prevented biofilm formation at concentrations close to the respective MICs, whereas rifampicin required concentrations 4–8 × higher than the MIC to achieve similar inhibition (Figure 1 and Supporting Information Table S2). CorA also efficiently eradicated established biofilms in concentrations close to its MIC, indicating efficient killing of biofilm‐embedded cells (Figure 1). Conversely, dalbavancin‐challenged isolates exhibited MBECs of at least 4–8× the MIC, but in some cases up to 128× above MIC (Figure 1 and Supporting Information Table S2), corroborating prior findings of suboptimal eradication of mature biofilms by the latter antibiotic in vitro [28]. These results underscore that dalbavancin, despite its long half‐life and lipophilicity, remains more effective at preventing biofilm formation than at eradicating established communities, a limitation shared with most glycopeptides [29]. Rifampicin displayed a distinct response pattern: despite being highly active against planktonic bacteria, it showed a lower efficacy against mature biofilms and required concentrations clearly exceeding the MIC to achieve measurable effects (Figure 1). This discrepancy aligns with earlier observations of diffusion limitations of rifampicin. The rapid development of rifampicin resistance, alongside its hepatotoxicity upon long‐term treatment and significant drug–drug interactions, further constrains its use in chronic infections [8, 29]. The elevated concentrations of dalbavancin and rifampicin required to inhibit biofilm formation and eradicate established biofilms are in line with observations for other antibiotics, which typically require several‐fold higher concentrations than the MIC due to limited penetration into the biofilm matrix, altered metabolic states of sessile bacteria, and high local cell densities [30, 31]. Notably, CorA maintained both inhibitory and eradication activities against rifampicin‐resistant isolates (Supporting Information Table S2). This dual advantage, low MBEC/MIC ratio and retained efficacy against rifampicin‐resistant strains, positions CorA as a promising candidate for managing biofilm‐associated infections, where established agents often fail due to diffusion barriers or rapid adaptation.

### 2.2 CorA Efficiently Reduces the Viable Population in Established Biofilms Comparable to Rifampicin

**FIGURE 2:**
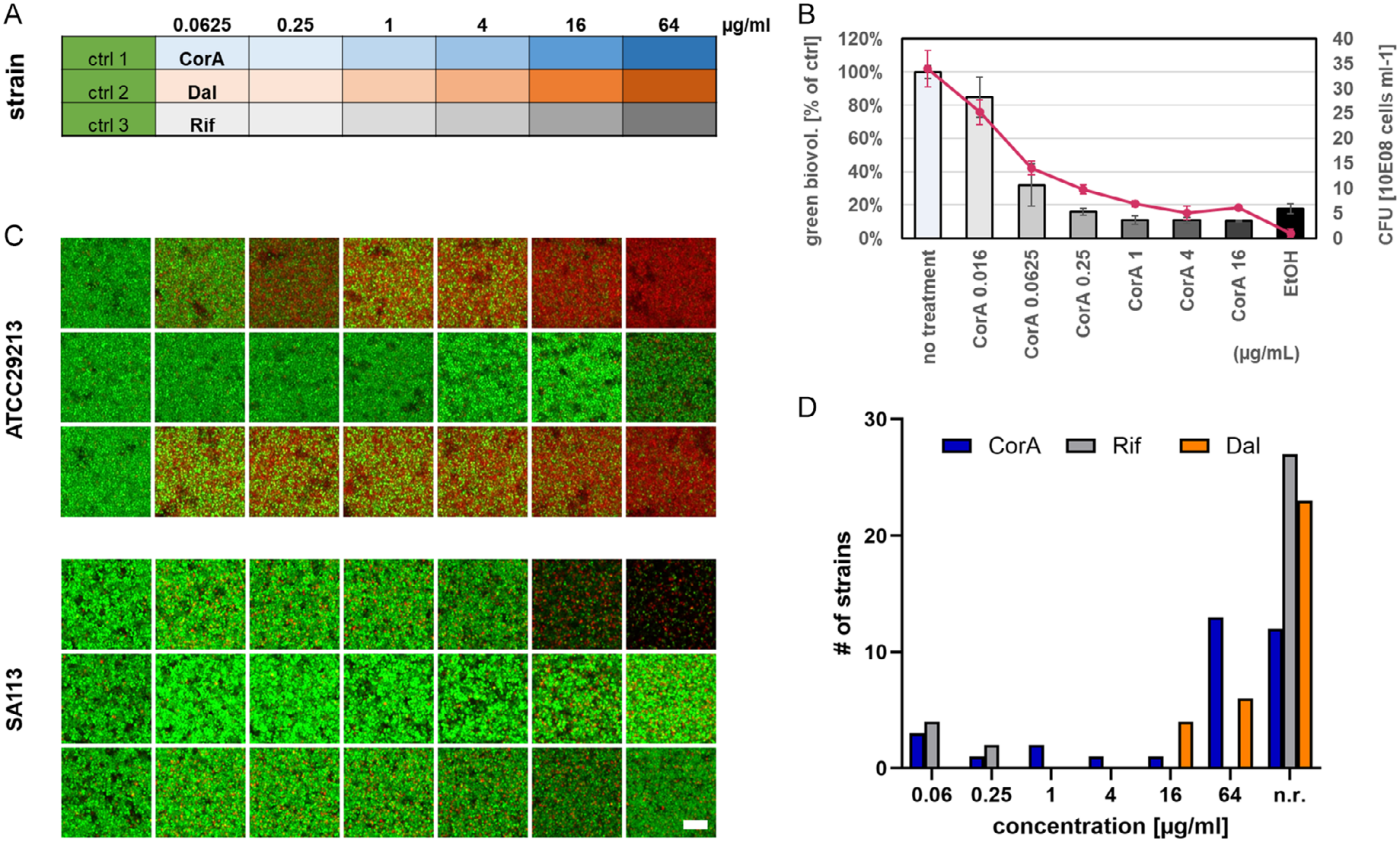
Results of the CLSM biofilm assay. (A) Experimental layout within the microtiter plate including concentration series of the drugs CorA, dalbavancin (Dal) and rifampicin (Rif) and three growth controls. (B) Correlation of BacLight viability staining and CFU counts including 25% EtOH as killing control. Primary *Y*‐axis represents the amount of relative green biovolume of the MSSA strain ATCC 29213 biofilm compared to the untreated control. Secondary *Y*‐axis represents the number of bacteria plated from the treated and stained wells. (C) Two examples of treated and stained staphylococci biofilms visualized as top‐down projections. Living cells are stained in green (Syto9), dead cells are stained in red (propidium iodide). Top‐down projections of all 33 strains can be seen in Supporting Information Figure S3. Scale bar 10 µm. (D) Summary of the capacity of the drugs to reduce the viable biovolume ≥75% of all 33 strains compared to the untreated control in dependence of the used antibiotic concentration. n.r., not reached.

To confirm the antibiotic efficacy of CorA towards established biofilms seen with the Calgary device, we adapted a previously established microscopic assay for testing against *S. aureus* biofilms [32]. The assay is based on the combination of BacLight viability staining and confocal laser scanning microscopy enabling the quantification of killing efficacies of the drugs rifampicin and dalbavancin in comparison to CorA. Since drugs were applied to 24 h old, established biofilms, higher drug concentrations were included in the assay (see layout Figure 2A) because far higher cell numbers (2–3 *log*‐scales) are expected in established biofilms compared to cell numbers used in MIC assays, which strongly impacts on antibiotic effectiveness of various drugs [33, 34]. As exemplarily tested for the strain ATCC 29213 in presence of increasing concentrations of CorA, there is a good correlation of viability staining and CFU counts (Figure 2B).

In total, we received valid image analysis data sets for the biofilms of 33 strains (28× *S. aureus*, 3× *S. epidermidis*, 1× *S. haemolyticus* + 1× *S. hominis*) summarized in Supporting Information Table S3. The strain collection included the strains used in the MBEC assay (except HG001), as well as additional laboratory strains. Since the evaluation of biofilm formation in the microscopic assay is complementary to the crystal violet assay and relies on biofilm growth at the bottom of a plate without prior washing steps, additional strains, not previously characterized as strong biofilm formers, were included. Top‐down projection of biofilm image z‐stacks – exemplarily visualized for the strain ATCC 29213 and SA113 in Figure 2C—were used to determine the reduction capacity of the three tested drugs by calculating the percentage of the living biofilm biovolume after 24 h treatment compared to the untreated control. As seen in Figure 2D, CorA reduced the living biovolume >75% for 64% of the strains of the selected panel (21/33), while rifampicin was only active against 18% of the strains (6/33), and dalbavancin against 30% (10/33). Our findings that dalbavancin was only able to reduce living biofilm biovolume at very high concentrations (16 and 64 µg/ml) is in line with the MBEC data (Figure 1) and its overall weak potential to remove established biofilms [26]. In the microscopic assay, it might also be a result of missing cation‐adjustment in the biofilm growth medium [35]. Since the test panel included 4 Rif‐R strains, it is likely that the rifampicin results are underestimating the biofilm reduction potential of this compound (without the 4 Rif‐R strains: 21% (6/29)). On the other hand, it nicely shows that CorA is also active against Rif‐R strains (Supporting Information Table S3) despite targeting the same bacterial enzyme.

Within our microscopic biofilm assay, CorA and rifampicin showed superior activity towards *S. aureus* biofilms compared to dalbavancin at low drug concentrations (MIC breakpoint levels of rifampicin 0.0625 µg/ml & dalbavancin 0.125 µg/ml). However, CorA showed better biofilm reduction at higher test concentrations (0.25 – 64 µg/ml), while rifampicin seemed to be either active or not active, which appears more obvious if we look at the lower reduction levels towards biofilm biovolume >25% and >50% (Supporting Information Figure S2). In the graph displaying the moderate biovolume reduction capacities of the drugs (≥25%), dalbavancin was active against more than 85% of the tested strains (28/33), but mainly at concentrations >1 µg/ml, while rifampicin was less active (73%, without the 4 Rif‐R strains (24/29)). CorA was active against 97% (32/33) of the strains. The only strain which was not affected by CorA was an *S. epidermidis* strain.

### 2.3 CorA Demonstrates Biofilm Prevention Capacities Comparable to Rifampicin in a *S. aureus* SA113‐Related Foreign Body Infection Model

**FIGURE 3:**
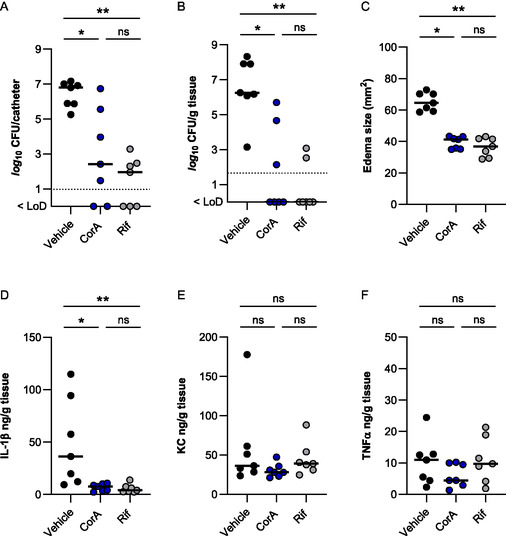
Efficacy of CorA in preventing biofilm formation in an *S. aureus* related murine foreign body infection model. The infection was established using a bacterial inoculum of 1 × 10^4^ colony forming units (CFU) of *S. aureus* strain SA113 into the lumen of the implanted catheter tubing fragment (1 cm). CorA (36 mg/kg) was administered subcutaneously twice a day (BID), starting at 3 h post infection. Untreated mice received an equal volume of the vehicle (80% PEG400/PBS) BID, while rifampicin (12.5 mg/kg BID) was used as a reference antibiotic. A‐C) At 6 days post infection, mice were euthanized, edema sizes around the implanted catheter fragments determined, and the catheter fragments and surrounding peri‐implant tissues explanted. Bacterial loads from catheter‐detached biofilms (A) and in the peri‐implant tissues (B) were determined by CFU counting. C) Edema sizes at 6 days post infection. D‐F) Interleukin‐1β (IL‐1β; D), keratinocyte chemoattractant (KC; E), and tumor necrosis factor alpha (TNFα; F) levels in peri‐implant tissue homogenates obtained at 6 days post infection. The data represent the value of every individual animal (symbols; *n* = 7 per group) with the median (continuous horizontal lines). Dashed horizontal lines indicate the limit of detection (LoD). *, *p* < 0.05; **, *p* < 0.01; ns, not significant (Kruskal–Wallis test followed by Dunn's post hoc test).

Given the promising anti‐biofilm activities of CorA against staphylococci in vitro, we next determined the biofilm prevention capacities of this compound in a murine foreign body related infection model [36, 37]. In this model, a 1 cm peripheral venous catheter fragment is implanted subcutaneously (s.c.) into the flank of the mouse and the catheter fragment subsequently infected with 10^4^ CFU of the biofilm‐forming *S. aureus* strain SA113. Treatment with CorA (36 mg/kg body weight s.c.) was started 3 h post infection and repeated twice per day for 6 days. This treatment regimen resulted in a significant reduction of the bacterial loads on the catheter fragment and in the peri‐implant tissue that formed around the catheter fragment when compared to the vehicle‐treated controls (Figure 3).

Specifically, treatment with CorA led to *a* > 4‐*log*_10_ reduction in median bacterial load found on the catheter fragments (4.4‐*log*_10_) and the peri‐implant tissues (6.3‐*log*_10_) formed around the catheter fragments, respectively. Notably, median CFU rates recovered from catheter fragments and peri‐implant tissues of CorA‐treated mice were on the same levels as those seen in mice treated with 12.5 mg/kg body weight s.c. of the reference antibiotic rifampicin twice per day (Figures 3A,B). Similarly, edema sizes recorded at the implant sites of CorA‐treated mice at day 6 post infection were significantly lower than those seen with vehicle‐treated mice and on a comparable level to those observed in the rifampicin‐treated mice (Figure 3C). To gain further insights into the humoral immune response of the infected and treated animals, the cytokine levels of interleukin 1β (IL‐1β), keratinocyte chemoattractant (KC), and tumor necrosis factor alpha (TNFα) were determined in the peri‐implant tissues harvested at 6 days post infection (Figure 3D–F). For IL‐1β, lower tissue levels were observed in rifampicin and CorA‐treated mice when compared to sham‐treated mice (Figure 3D), while tissue levels for KC (Figure 3E) and TNFα (Figure 3F) were comparable for all three groups. Taken together, these findings suggest that treatment with CorA is comparable to rifampicin treatment in reducing the bacterial population formed by *S. aureus* on an implanted medical device, in diminishing the dissemination of the bacteria into the surrounding tissue, and in suppressing the expansion of the inflammation at the infection site. Finally, this positive proof‐of‐concept paves the way for further assessment of CorA as a treatment option against biofilm‐associated infections with staphylococci. Given the strong ability of CorA to penetrate bone tissue [21], additional potential applications, such as treating staphylococcal osteomyelitis, may be explored in the future.

## 3 Conclusion

This study demonstrated the capacity of CorA to inhibit the biofilm formation of and to eradicate preformed staphylococcal biofilms both in vitro and in vivo. The in vitro assays revealed that CorA was effective against biofilm from nearly all tested strains, outperforming current antibiotics in clinical use, *i.e.* dalbavancin and rifampicin. Importantly, in a murine foreign body infection model, CorA achieved the reduction in bacterial load and inflammation similar to rifampicin, one of the current standard options in these indications. This underlines its potential for the treatment of device‐related infections and renders CorA a promising therapeutic candidate. Therefore, future in‐depth investigations mimicking distinct clinical scenarios will aim to qualify CorA for targeting staphylococcal biofilm‐associated infections beside its primary indication against filarial diseases.

## 4 Experimental Section

### 4.1 Bacterial Strains

All bacterial strains used were kindly provided by Prof. Gabriele Bierbaum (University Hospital Bonn, Germany).

### 4.2 Compounds

CorA with a purity > 91% was produced using the heterologous producer strain *Myxococcus xanthus* carrying the CorA biosynthetic gene cluster and purified as previously reported [19, 38, 39].

Dalbavancin was purchased from Biomol (Hamburg, Germany) and dissolved in 100% (v/v) DMSO to a stock concentration of 2 mg/mL. For the determination of MIC, MBIC, and MBEC, rifampicin was obtained from Merck (Darmstadt, Germany) and resuspended in sterile water for injection to a concentration of 0.5 mg/mL. For the viability assays, rifampicin was purchased from Roth (Karlsruhe, Germany).

### 4.3 Determination of the MIC via the Broth Microdilution Method

The minimum inhibitory concentration (MIC) was determined using the broth microdilution method according to the EUCAST protocols [40], with small modifications. Briefly, the bacterial inoculum was prepared by adjusting the turbidity of an overnight culture to the 0.5 McFarland standard using sterile saline. This solution was then diluted 1:100 in tryptic soy broth (TSB; Merck, Germany). Serial twofold dilutions of the antibiotics (16– 0.0078 µg/ml for CorA and dalbavancin, 1–0.00049 µg/ml for rifampicin) were prepared in 50 µl of TSB in a sterile 96‐well microtiter plate. In the case of dalbavancin, the surfactant Tween80 (0.002% v/v) was also added to the medium. Each well was inoculated with 50 µl of the diluted bacterial suspension to reach a final volume of 100 µL per well. Positive growth controls (TSB with bacteria but no antibiotic) and sterility controls (TSB only) were included on each plate. The plates were incubated statically at 37°C for 18–24 h. After incubation, wells were visually inspected for turbidity, and the MIC was defined as the lowest concentration of antibiotic at which no visible bacterial growth was observed. All assays were performed in triplicate to ensure reproducibility.

### 4.4 Characterization of Biofilm Using Crystal Violet Staining

Biofilm formation was determined in a 96‐well plate format with adaptation from the previously published paper [25]. Briefly, a staphylococcal overnight culture was diluted 1:100 in TSB supplemented with 0.5% (w/v) glucose (TSB‐G) to a volume of 200 µl/well in a flat bottom 96‐well plate (Thermo Scientific, Denmark) and grown for 24 h without shaking at 37°C. After the incubation, wells were washed twice with 200 µl of phosphate buffered saline (PBS, pH 7.4) to remove planktonic cells, fixed with 95% ethanol for 5 min, and stained with 1% (w/v) crystal violet for 5 min. Excess dye was removed by washing the wells twice with water, and the biofilm‐bound dye was solubilized with 30% (v/v) acetic acid for 10 min. Biofilm formation was quantified by measuring the absorbance at 560 nm.

### 4.5 Determination of the MBIC in the Calgary System

Biofilm susceptibility testing was performed using the standard Calgary Biofilm Device (Innovotech Inc., Edmonton, Canada) following the MBEC Assay manual with minor modifications [26]. Briefly, serial twofold dilutions of the antibiotics from 8× to 0.0625× MIC were prepared in 100 µl of TSB‐G in a sterile 96‐well microtiter plate. In the case of dalbavancin, the surfactant Tween80 (0.002% v/v) was also added to the medium. One hundred microliters of the bacterial suspension (∼5 × 10^5^ CFU/mL, prepared from a culture in log‐phase) in TSB‐G were added. The plate also included at least one column of sterile control and two columns of untreated growth controls for each strain. Each compound concentration was determined in triplicate in each plate. The plates were covered with the 96‐peg lid and biofilms were allowed to grow in the presence of the antibiotics, statically, at 37°C in a humidity‐controlled environment for 24 h. At the end of the incubation time, biofilm‐coated pegs were gently rinsed in 200 µl saline solution to remove planktonic and loosely attached cells and transferred to a recovery plate containing 200 µL of TSB. Biofilms were removed by sonication in an Emerson Bransonic M mechanical bath 5800 at max intensity for 30 min to release adherent cells. The plates were then covered with a sterile plastic lid and incubated at 37°C until the drug‐free growth controls reached OD_600_ 0.5–0.6. Optical density at 600 nm (OD_600_) was then measured to quantify bacterial recovery. The MBIC was defined as the concentration that resulted in greater than 75% inhibition of the biofilm formation compared to the drug‐free growth control.

### 4.6 Determination of the MBEC in the Calgary System

The MBEC was also determined using the Calgary Biofilm Device, with the difference that the biofilm was established prior to the challenge with the antibiotics. In this case, 200 µl of bacterial suspension (∼5 × 10^5^ CFU/mL, prepared from a culture in *log*‐phase) in TSB‐G were added to a sterile 96‐well plate, leaving at least one column of sterile control. Plates were incubated statically at 37°C in a humidity‐controlled environment for 24 h to allow biofilm formation. Afterwards, the biofilm‐coated pegs were gently rinsed in 200 µl saline solution to remove planktonic and loosely attached cells and then transferred to a challenge plate containing test compounds prepared as twofold serial dilutions from 128× to 0.125× the MIC (final volume 200 µl). The plate also included two columns of untreated growth controls for each strain. Following 24 h incubation at 37°C, the pegs were briefly rinsed and transferred to a recovery plate containing 200 µL of TSB. The following steps were the same as for the MBIC determination. The MBEC was defined as the concentration that resulted in greater than 75% reduction of the biofilm, when compared to the growth control.

### 4.7 Determination of the Viable Biofilm Population via Confocal Laser Scanning Microscopy

The method was adapted from the previously established for *Pseudomonas aeruginosa* [32]. In brief, staphylococci precultures were diluted 1:100 in Muller‐Hinton medium (CMB405B, Oxoid/Thermo Fisher Scientific; providing the best biofilm results in this assay), and 100 µl were used as inoculum to establish bacterial biofilms in a μclear half area plate (Greiner Bio‐one) for 24 h at 37°C in a humid atmosphere. After 24 h, antibiotics were added to the wells. Antibiotic (Rif/Dal/CorA) concentrations: 0.0625/0.25/1/4/16/64 µg/ml. Solvent control: 1% DMSO, killing control: 25% ethanol. The BacLight Viability kit (Molecular Probes, Inc.) was added (final dilution 1:1600) simultaneously to differentiate live (Syto9) and dead (propidium iodide) bacteria. Biofilms were further incubated for 24 h followed by analysis via confocal laser‐scanning microscopy (CLSM). Automated image acquisition of biofilm z‐stacks was done with an inverted SP8 system (Leica Microsystems) equipped with na 40×/NA 1.1 water objective and operated via the Leica application suite LAS X including the Matrix screener module. For each well, two image stacks were acquired in sequence at the center of each well with two magnifications: stack 1 (overview job) was acquired using a zoom × 0.75 (image size: ∼387 × 387 μm, pixel size of 0.378 μm) and a total range of ∼ 40 μm (20 slides and a slice distance of 2 μm), while stack 2 (zoom job) was acquired with a zoom × 8 (image size: ∼37 μm × 37 μm; pixel size of 0.071 μm) to visualize single cells. For Syto9, we used the 488 nm laser (0.5%) and an emission range of 500–550 nm; for PI, we used the 561 nm laser (15%) and an emission range 650–750 nm. To compensate for interspecies and interwell fluctuations, a number of predefined PMT detector settings were assigned for the different strains/isolates tested. The two acquired image stacks were analyzed with the Developer XD (Definiens) software to determine dye‐ and biofilm‐specific parameters including, for example, the ratios of the differentially stained populations within the biofilm or the biovolume describing the biofilm biomass. For final analysis, the data of both image stacks was combined to determine the percentage of biofilm reduction of the living population in comparison to the control. For the categorization within one group (biofilm reduction: >25%, >50%, >75%), all higher antibiotic concentration within one test combination (1 strain+1 antibiotic) had to display the same or higher reduction values. For CFU counts, biofilms of the optical plate were vigorously pipetted, diluted in 1:10 steps and platted to count the number of colonies on agar plates.

### 4.8 *S. aureus*‐Related Murine Foreign Body Infection Model

Animal experiments were performed with approval of the local State Review Board of Saarland, Germany (project identification code 14/2021 [approved 04.05.2021]), and conducted following national and European guidelines for the ethical and humane treatment of animals. Seven‐week‐old female C57BL/6N mice were obtained from Charles River (Sulzfeld, Germany) and kept under specific pathogen‐free conditions according to the regulations of German veterinary law. Mice were allowed to adapt to the environment for at least 7 days. TSB‐washed exponential growth phase cells (∼1 × 10^4^ CFU) of the polyintercellular adhesin producing *S. aureus* strain SA113 [41, 42] were used as inoculum. Implantation of catheter fragments and infection of animals was carried out essentially as described [36], except that only implanting one catheter fragment (BD Venflon Pro Safety 14 G, BD, Heidelberg, Germany) per mouse. Starting at 3 h post infection, mice were treated with ∼50 µL of CorA (14.4 mg/mL, equivalent to 36 mg of CorA per kg body weight) dissolved in a PEG400/PBS (80:20 v/v) solution, which was injected subcutaneously into the catheter loaded flank, repeated every 12 h. Mice treated either with ∼50 µL of rifampicin (5 mg/mL; Sigma–Aldrich SBR00067, Merck, Darmstadt, Germany; equivalent to 12,5 mg/kg body weight) dissolved in physiological saline solution or with vehicle alone (PEG400/PBS (80:20 v/v)) served as controls. Behavior and weight of the animals were monitored daily. Six days post infection, animals were euthanized, edema sizes were measured, and catheter fragments with surrounding tissue were harvested for microbial analyses as described before [36].

### 4.9 Peri‐Implant Tissue Cytokine Determinations

Enzyme‐linked immunoadsorption assays were performed essentially as described [43]. The peri‐implant tissue homogenates were centrifuged at 2,500 × g for 10 min at 4°C to remove cellular debris. To obtain a high‐quality cell‐free sample, the resulting supernatant was subjected to a second centrifugation at 15,000 × g for 15 min. The final supernatants were carefully collected to ensure debris‐free samples and subsequently stored at –70°C. Quantification of the pro‐inflammatory cytokines IL‐1β, KC, and TNF‐α in the tissue homogenates was performed using R&D ELISA kits (No. DY401, DY453, and DY410; R&D Systems; Fisher Scientific, Nidderau, Germany) according to the manufacturer's protocol. Briefly, 96‐well microplates were coated with the respective capture antibody and incubated at room temperature overnight. The following day, the wells were blocked with reagent diluent (1% BSA solution) for 2 h, followed by sequential incubations with samples and detection antibody (2 h) and streptavidin‐HRP (20 min). The reaction was initiated by adding the BM Blue POD substrate solution and stopped after 20 min with 2N H_2_SO_4_. The absorbance was measured at 450 nm with background subtraction at 540 nm. A serially diluted standard was run in parallel. Samples were diluted with reagent diluent so that their absorbance values fell within the range of the standard curve. Protein concentrations were normalized to tissue weight.

## Supporting Information

Additional supporting information can be found online in the Supporting Information section. **Supporting Fig. S1**: Comparison between the OD_600_ measurements (horizontal lines) and the CFU/ml determination (solid) in the destruction of *S. aureus* ATCC 53657‐A with CorA (blue, A) and Rif (grey, B) and ATCC 25923 with CorA (blue, C) and Rif (grey, D). The MBEC is determined at the fold concentration where less than 25% of the biofilm is remaining, compared to the growth control (dotted line). Mean of three replicates is shown. Error bars indicate standard deviation. **Supporting Fig. S2**: Summary of the capacity of the drugs to reduce the viable biofilm biovolume about ≥25% (A) and ≥50% (B) compared to the untreated control and in dependence of the used antibiotic concentration. In all three graphs (+ Figure 2D) all 33 strains are included. n.r., not reached. **Supporting Fig. S3**: Top‐down projections of CLSM z‐stacks of treated and stained staphylococci biofilms. Living cells are stained in green (Syto9), dead cells are stained in red (propidium iodide). Single images have a size of 37 μm * 37 μm (zoom job). Montage images have been contrast adjusted for better visualization of all images. Plate layout same as in Figure 2A. **Supporting Table. S1**: Biofilm formation and resistance phenotype of the tested *S. aureus* (A) and CoNS (B) strains. Values correspond to the absorbance at 560 nm measured after crystal violet staining on 24 h biofilms. **Supporting Table. S2**: MIC, MBIC, and MBEC values of the 15 selected strains determined with the Calgary device. **Supporting Table. S3**: Minimal antibiotic concentrations needed to reduce the viable biofilm biovolume by about ≥25% / ≥50% / ≥75% compared to the untreated control.

## Funding

This study was supported by the Deutsches Zentrum für Infektionsforschung (TTU‐09.921) and the TALENTS Marie Skłodowska‐Curie COFUND‐Action of the European Union (101081463).

## Conflicts of Interest

The authors declare no conflicts of interest.

## Data Availability

The data that support the findings of this study are available from the corresponding author upon reasonable request.
